# Diabetic dyslipidemia impairs coronary collateral formation: An update

**DOI:** 10.3389/fcvm.2022.956086

**Published:** 2022-08-22

**Authors:** Ying Shen, Xiao Qun Wang, Yang Dai, Yi Xuan Wang, Rui Yan Zhang, Lin Lu, Feng Hua Ding, Wei Feng Shen

**Affiliations:** ^1^Department of Cardiovascular Medicine, School of Medicine, Ruijin Hospital, Shanghai Jiao Tong University, Shanghai, China; ^2^Shanghai Clinical Research Center for Interventional Medicine, Shanghai, China; ^3^Institute of Cardiovascular Disease, Shanghai Jiao Tong University School of Medicine, Shanghai, China

**Keywords:** dyslipidemia, type 2 diabetes mellitus, coronary collateral circulation, coronary artery disease, lipid-lowering therapy

## Abstract

Coronary collateralization is substantially impaired in patients with type 2 diabetes and occlusive coronary artery disease, which leads to aggravated myocardial ischemia and a more dismal prognosis. In a diabetic setting, altered serum lipid profiles and profound glycoxidative modification of lipoprotein particles induce endothelial dysfunction, blunt endothelial progenitor cell response, and severely hamper growth and maturation of collateral vessels. The impact of dyslipidemia and lipid-lowering treatments on coronary collateral formation has become a topic of heightened interest. In this review, we summarized the association of triglyceride-based integrative indexes, hypercholesterolemia, increased Lp(a) with its glycoxidative modification, as well as quantity and quality abnormalities of high-density lipoprotein with impaired collateral formation. We also analyzed the influence of innovative lipid-modifying strategies on coronary collateral development. Therefore, clinical management of diabetic dyslipidemia should take into account of its effect on coronary collateralization in patients with occlusive coronary artery disease.

## Introduction

Type 2 diabetes mellitus (T2DM) is increasingly prevalent worldwide, and cardiovascular disease represents the leading cause of morbidity and mortality in patients with T2DM (1). A cluster of lipid metabolic abnormalities, collectively referred to as diabetic dyslipidemia, have been well established as a major risk factor for adverse cardiovascular outcomes in diabetic patients. The pattern of diabetic dyslipidemia consists of hypertriglyceridemia associated with increased triglyceride-rich lipoproteins and their remnants, decreased high-density lipoprotein cholesterol (HDL-C), and elevated low-density lipoprotein cholesterol (LDL-C) levels with predominance of small dense LDL-C (sdLDL-C). Meanwhile, hyperglycemia and chronic inflammation in diabetic conditions promote glycation and oxidative modification of lipoprotein particles, leading to changes in conformation and function, altered interaction with membrane receptors and downstream signaling, and switch of the phenotype toward a more atheroprone state (2).

Coronary collaterals have been recognized as an important compensatory mechanism in salvage of ischemic myocardium, preservation of left ventricular function, and improvement of prognosis for patients with obstructive coronary artery disease (CAD) (3–5). During the development of coronary collaterals, two distinct processes, arteriogenesis and angiogenesis, are involved. The former pertains to the remodeling of preexisting arterial vessels through anatomic increase in lumen area and wall thickness. The latter is defined as growth of new capillaries that stem from the budding of preexisting capillary vessels (6). These processes are finely tuned by a variety of biomechanical and biochemical factors, including perfusion pressure, wall shear stress, systematic hypoxia, oxidative stress, inflammatory response and endothelial function.

Numerous clinical observations reveal substantially impaired collateral circulation in occlusive CAD patients with diabetes. Given the generally more severe atherosclerotic lesions and microcirculation dysfunction in diabetic patients, poor collateralization may provide a significant add-on effect to aggravate myocardial ischemia and contribute to a more dismal prognosis (7). A couple of underlying mechanisms for the poor collateral formation in diabetic patients have been identified. Chronic hyperglycemia and the engagement of advanced glycation end-products with their receptors (AGE-RAGE axis) adversely affects collateral development by inhibiting vessel growth and maturation (8). On the other hand, disturbed lipid metabolism also plays a critical role and is regarded as a hallmark of impaired angiogenesis (9, 10). Currently, the impact of dyslipidemia and lipid-lowering treatments on coronary collateral formation has become a topic of heightened interest. This review is the first to summarize the recent literature, in combination with our study findings, to elucidate the association of different components of diabetic dyslipidemia with coronary collateralization and highlight their potential clinical implications in T2DM patients with CAD. The relevant clinical studies investigating the association between lipid profiles and coronary collateralization are summarized in Table 1.

**TABLE 1:** Summary of clinical studies investigating the association between lipid profiles and coronary collateralization.

Authors	Study population	Design	Number of patients	Parameter of lipid profiles	Main relevant results
Liu et al. (10)	Consecutive patients with CTO undergoing CAG	Observational study	1,653 patients (poor CC: 355; good CC: 1298)	TG	After multiple adjustment, the quartiles of TG (adjusted OR = 1.267, 95% CI 1.088–1.474, *P* = 0.002) remained an independent factor of poor CC.
Gao et al. (16)	Consecutive patients with acute coronary syndrome and CTO	Observational study	1,093 patients (poor CC: 775; good CC: 318)	TyG index	TyG index was significantly higher in patients with poor CC compared to those with good CC (9.3 ± 0.65 vs. 8.8 ± 0.53, *P* < 0.001). The proportion of poor CC increased stepwise from the lowest to the highest TyG index tertile (15.3% vs. 22.8 % vs. 49.2 %, *P* < 0.001). After adjusting for confounding factors, TyG index remained correlated with the occurrence of impaired CC (OR 1.59, 95% CI 1.07–2.36 and OR 5.72, 95% CI 3.83–8.54 for middle and highest tertile groups vs. lowest tertile group, all *P* < 0.001) The improvement of the AUC for assessing poor CC was most significant when adding TyG index to baseline model, with a best cut-off value of 9.105. The most significant enhancement in risk reclassification and discrimination was found after inclusion of TyG index into baseline model, with a NRI of 0.238 (*P* < 0.001) and an IDI of 0.103 (*P* < 0.001).
Liu et al. (18)	Consecutive patients (≥60 years) with ST-elevation MI undergoing primary PCI	Retrospective case-control study	346 patients (poor CC: 238; good CC: 108)	TG/HDL	TG/HDL ratio was significantly higher in patients with poorly developed CC than in those with well-developed CC (2.88 ± 2.52 vs. 1.81 ± 1.18, *P* < 0.001). In multivariate logistic regression analysis, higher TG/HDL ratio served as an independent positive predictor of poor development of CC (OR 1.789, 95% CI 1. 346–2.378, *P* < 0.001). The AUC of TG/HDL ratio for predicting poor CC was 0.716 (95% CI 0.654–0.778, *P* < 0.001) with an optimal cut-off value of 1.58, sensitivity of 55.7% and specificity of 71.9%.
Aras et al. (33)	Stable angina pectoris with CTO of one major coronary artery	Retrospective study	60 patients (poor CC: 31; good CC: 29)	Lp(a)	Lp (a) levels were significantly higher and vascular endothelial growth factor levels were significantly lower in patients with poor CC than in those with good CC (34 ± 19 vs. 20 ± 12 mg/dl, *P* < 0.001, and 2.5 ± 0.7 vs. 3.4 ± 0.8 ng/dl, *P* < 0.001, respectively). Poorly developed CC were more prevalent in patients with Lp (a) levels ≥30 mg/dl than in those with Lp (a) levels <30 mg/dL (72 vs. 37%, *P* = 0.008). A strong negative correlation was observed between Lp (a) and vascular endothelial growth factor (*r* = −0.708, *P* < 0.0001). High levels of Lp (a) negatively affected the development of CC (adjusted OR 0.92, 95% CI 0.88–0.96, *P* = 0.009).
Fan et al. (34)	Chronic stable coronary disease with at least one major coronary occlusion or a stenosis of ≥95% with TIMI grade 1	Observational study	654 patients (Rentrop score 0, 1, 2, and 3 in 44, 91, 232, and 287 patients, respectively)	Lp(a)	Lp(a) levels were significantly decreased across Rentrop score 0–3 (25.80 ± 24.72, 18.99 ± 17.83, 15.39 ± 15.80, and 8.40 ± 7.75 mg/dL, *P* < 0.001). In model 1, the risk of poor CC (Rentrop 0) was greater in the third Lp (a) tertile compared to the first Lp(a) tertile (OR 3.34, 95% CI 2.32–4.83, *P* < 0.001). In model 2, the risk of poor CC (Rentrop 0) was greater in Lp(a) >30 mg/dL group compared to Lp(a) <30 mg/dL group (OR 6.77, 95% CI 4.44–10.4, *P* < 0.001).
Shen et al. (35)	Consecutive stable CAD patients with CTO of at least one major epicardial coronary artery	Observational study	1284 patients (DM: 706; non-DM: 578; poor CC: 505; good CC: 779)	Lp(a) TC LDL-C Non-HDL HDL-C TG	For diabetic and non-diabetic patients, Lp(a), total cholesterol, LDL-C, and non-HDL-C levels were higher in patients with poor CC than in those with good CC, whereas HDL-C and TG levels were similar. After adjustment for potential confounding factors, tertiles of Lp(a), total cholesterol, LDL-C and non-HDL-C remained independent determinants for poor CC. A significant interaction between Lp(a) and total cholesterol, LDL-C or non-HDL-C was observed in diabetic patients (all P interaction <0.001) but not in non-diabetics. At high tertile of total cholesterol (≥5.35 mmol/L), LDL-C (≥3.36 mmol/L) and non-HDL-C (≥4.38 mmol/L), diabetic patients with high tertile of Lp(a) (≥30.23 mg/dL) had an increased risk of poor CC compared to those with low tertile of Lp(a) (<12.66 mg/dL) (adjusted OR = 4.300, 3.970 and 4.386, respectively, all *P* < 0.001).
You et al. (36)	Consecutive acute MI undergoing interventional CAG	Observational study	409 patients (poor CC: 277: good CC 132)	Lp(a)	Patients with poor CC had a higher Lp (a) level than those with good CC (219.1 [98.0–506.9] vs. 122.0 [64.5–215.6] mg/L, *P* < 0.001). The AUC of Lp(a) for predicting poor CC was 0.647 (95% CI: 0.592–0.702) with the cut-off value of 199.0 mg/L, sensitivity of 55.7% and specificity of 71.9%. Regression analyses revealed that patients with high Lp(a) levels had a greater risk of poor CC than those with low Lp(a) levels (unadjusted OR 2.924, 95% CI 1.900–4.501; adjusted OR 2.929, 95% CI 1.863–4.604, both *P* < 0.001). Patients with Lp(a) ≥30 mg/dL also had a greater risk of poor CC than those with Lp(a) <30 mg/dL (unadjusted OR 3.394, 95% CI 2.042–5.640; adjusted OR 4.232, 95% CI 1.400–12.797, both *P* < 0.001).
Kadi et al. (38)	Consecutive patients with CTO of at least one major epicardial coronary artery	Case-control study	151 patients (poor CC: 49; good CC: 102)	HDL-C	Serum HDL-C was lower in poor CC group compared to good CC group (34.9 ± 8 mg/dL vs. 43.7 ± 9.4 mg/dL, *P* < 0.001). The proportion of patients with low HDL-C was significantly higher in the poor CC group compared with the good CC group (*P* < 0.001). Serum TG levels and percentage of MI history were higher in the poor CC group compared with good CC group (*P* = 0.015 and *P* = 0.026, respectively). There was a positive and strong correlation between Rentrop grade and serum HDL-C level (*r* = 0.503, *P* < 0.001). Multivariate regression analysis showed that reduced HDL-C level was an independent predictor for poor CC (OR 4.3, 95% CI 1.964–9.369, *P* < 0.001).
Hsu et al. (39)	Consecutive patients undergoing CAG	Case-control study	501 patients (poor CC: 311; good CC:190)	HDL-C	There was no significant difference in HDL-C and other variables between good and poor CC. Multivariate analysis showed only number of diseased vessels was a significant predictor of poor collateral development (OR 0.411, *p* < 0.001).
Lee et al. (44)	Consecutive patents undergoing CAG	Case-control study	226 patients (poor CC: 71; good CC:155)	CEC	CEC was higher in the good than in the poor CC group (22.0 ± 4.6% vs. 20.2 ± 4.7%, *P* = 0.009). In multivariable analyses, CEC was identified as an independent predictor of good CC after adjustment for age, sex, HDL-C (OR, 1.10, 95% CI 1.03–1.18, *P* = 0.004). It remained significant after additional adjustment for DM, acute coronary syndrome, and Gensini score (OR 1.09, 95% CI 1.02–1.17, *P* = 0.011).
Wang et al. (46)	Patients with stable angina and angiographic CTO of at least one major coronary artery	Case-control study	437 patients (DM: 102; non-DM: 355; poor CC: 210; good CC: 227)	CEC	Compared to good collateralization group, CEC in poor collateralization group was significantly higher in non-diabetic patients (17.54 ± 11.86% vs. 13.91 ± 9.07%, *P* = 0.002). In contrast, CEC was impaired in type 2 diabetes irrespective of collateralization status (14.66 ± 10.47% vs. 13.26 ± 8.64%, *P* = 0.462). CEC correlated closely with Rentrop score in non-diabetic subjects, whereas no such association was present for HDL-C or apolipoprotein A-I. After adjusting for conventional risk factors including apolipoprotein A-I in logistic regression analysis, elevated CEC was independently associated with higher risk of poor collateralization in non-diabetic but not in diabetic subjects.

AUC, area under the curve; CAD, coronary artery disease; CAG, coronary artery angiography; CC: coronary collaterals; CEC, cholesterol efflux capacity; CI, confidence interval; CTO: chronic total occlusion; DM, diabetes mellitus; HDL-C, high-density lipoprotein cholesterol; IDI, integrated discrimination improvement; LDL, low-density lipoprotein; Lp(a): lipoprotein (a); MI: myocardial infarction; NRI, net reclassification improvement; OR, odds ratio; PCI, percutaneous coronary intervention; TC, total cholesterol; TG, triglyceride; TyG index, triglyceride-glucose index.

## Impact of diabetic dyslipidemia on collateral formation

### Hypertriglyceridemia

In patients with impaired glucose tolerance, blunt insulin sensitivity leads to compensatory hyperinsulinemia and increases secretion of triglyceride and triglyceride-rich lipoproteins. Hypertriglyceridemia confers an increased risk of CAD and adverse outcomes in patients with T2DM, by promoting release of excessive free fatty acids and stimulating production of proinflammatory cytokines, fibrinogen, and coagulation factors (11). Previous studies have shown that certain conditions with a cluster of risk components including hypertriglyceridemia (e.g., metabolic syndrome, overweight, or obesity) are more likely to be associated with endothelial dysfunction and reduced new vessel growth (10, 12), but the independent role of hypertriglyceridemia in coronary collateral formation remains difficult to be proven largely due to concomitant changes in other lipoproteins and relevant factors, particularly in patients with T2DM.

In recent years, several novel indexes by integrating triglyceride with some related metabolic measurements (such as HDL-C and glucose) have been proposed to better stratify the status of coronary collateralization. Triglyceride-glucose (TyG) index, calculated as log [fasting triglycerides (mg/dL) × fasting blood glucose (mg/dL)/2], has been suggested as a surrogate marker of insulin resistance (13). Elevated TyG index correlates well with high arterial stiffness and microvascular damage (14), which is associated with decreased coronary perfusion, reduced shear stress and arteriogenesis (15). In a large observational study, chronic total occlusion patients with poor coronary collaterals had higher TyG index compared to those with good collaterals. TyG was significantly associated with poor collateral formation even after adjusting for various confounders (16). Triglyceride to HDL-C ratio (TG/HDL-C ratio) and atherogenic index of plasma (logarithmic transformation of TG/HDL-C ratio) reflect the comprehensive situation of blood lipids and severity of insulin resistance (17). An observational study of elderly patients with acute myocardial infarction showed that an elevated TG/HDL ratio was independently associated with poor development of coronary collateral circulation (18). Of note, although a number of reports have suggested the prognostic role of atherogenic index of plasma beyond traditional risk factors (19, 20), further prospective studies are needed to examine if this index is applicable to predict coronary collateralization in type 2 diabetic patients with CAD.

### Hypercholesterolemia

Chronic exposure to high levels of cholesterol and LDL-C results in functional and structural abnormalities of the vasculature, including endothelial dysfunction, subendothelial lipid deposition, plaque progression and compromised collateral vessel growth (21, 22). In diabetic dyslipidemia, hypercholesterolemia and a predominant increase in sdLDL particles play negative roles in coronary collateral formation.

Under hypercholesterolemia, angiogenesis and arteriogenesis in response to tissue hypoxia are markedly attenuated. Hypercholesterolemia decreases endothelial nitric oxide (NO) bioavailability and NO synthase (eNOS) expression and activity which are essential for endothelial progenitor cell (EPC) migration (23). In animal models, cholesterol at high concentration resulted in delayed native arteriolar growth caused by reduced early monocyte/macrophage influx and migration, and even mildly elevated cholesterol significantly decreased expression of fibroblast growth factor (FGF) receptor 1, vascular cell adhesion molecule-1, and macrophage scavenger receptor-1, mimicking relative changes in arteriogenesis and tissue perfusion (24). The extent of these alterations was related to the duration of hypercholesterolemia. In patients with hypercholesterolemia, the number and activity of circulating EPCs were decreased compared to normocholesterolemic subjects (25). Circulating EPCs have special cellular machinery that is resistant to various types of stress, which allow them to participate in tissue repair. Interestingly, hypercholesterolemia reduced arteriogenesis more dominantly than hyperglycemia or hyperinsulinemia (26).

The inhibitory effect of hypercholesterolemia on angiogenesis/arteriogenesis could be attributed to the negative effect of LDL-C on endothelial cell responsiveness to growth factors (25). T2DM is usually accompanied by oxidation or glycation of LDL, and glycoxidatively modified LDL poses more pro-atherogenic and antiangiogenic properties than native LDL (27). In addition, the predominance of sdLDL-C confers a threefold increased risk for CAD, owning to their greater propensity for endothelial penetration into arterial wall, lower binding affinity for LDL receptor, longer circulation time, and higher susceptibility to glycation, oxidative modification, and uptake by scavenger receptors (28). The prospective Framingham offspring study and large cohort studies suggest that sdLDL is superior to LDL-C and other biomarkers in predicting future cardiovascular events in stable CAD patients with T2DM or hypertriglyceridemia (29–31). Nevertheless, there is a paucity of clinical data regarding the impact of elevated LDL-C and sdLDL-C on collateral formation in T2DM patients with CAD.

### Increased circulating lipoprotein(a)

Lipoprotein(a) is a genetically determined lipoprotein, which contains principally a cholesterol rich LDL particle, one molecule of apo B-100, and an apo (a). Noteworthy, Lp (a) is known to have atherothrombogenic property by inhibiting fibrinolysis system and promoting thrombus formation. In spite of a very skewed distribution, elevated circulating Lp(a) has emerged as an independent predictor of adverse outcomes for both general and higher risk populations, especially when LDL-C levels are elevated (32). In observational studies of patients with stable CAD, serum Lp(a) levels decreased stepwise across angiographic coronary collateral grade, and elevated Lp(a) predicted poor collateral development (33, 34). Intriguingly, a robust association between Lp(a) interactions with cholesterol-containing lipids and coronary collateral formation was suggested in patients with T2DM, which was non-linear and limited to high Lp(a) and LDL-C or non-HDL-C levels (35). At high tertiles of total cholesterol (≥5.35 mmol/L), LDL-C (≥3.36 mmol/L) and non-HDL-C (≥4.38 mmol/L), patients with high tertile of Lp(a) (≥30.23 mg/dL) had a significantly increased risk of poor collateralization compared with those with low tertile of Lp(a) (<12.66 mg/dL) (all *P* < 0.001). Furthermore, the additional inclusion of interaction of Lp(a) with total cholesterol, LDL-C and non-HDL-C provided better risk prediction of poor coronary collaterals. However, no interaction of Lp(a) with HDL-C and triglyceride on coronary collateralization was observed. In patients with acute myocardial infarction, increased Lp(a) in serum was closely correlated with poor coronary collaterals (36). Overexpression of Lp(a) in transgenic mice resulted in markedly reduced natural recovery of blood flow in hindlimb ischemia animal models in a dose-dependent manner. Lp(a) was found to stimulate the growth of vascular smooth muscle, which was reversed by intramuscular injection of hepatocyte growth factor (HGF) (37). Overall, these results highlight that Lp(a) may reflect coronary collateral status.

Lipoprotein(a) is highly concentrated in the arterial wall, carries cholesterol and binds oxidized phospholipids. Elevated circulating Lp(a) inhibits transforming growth factor-β activity and attenuates synthesis and/or release of vascular endothelial growth factor (VEGF) and decreases production of endothelium-derived NO, leading to impaired angiogenesis (33). Moreover, one of our ongoing studies indicates that circulating Lp(a) can also undergo glycation modification. As a characteristic protein of Lp(a), apo(a) is a large protein containing many kringle domains. Based on mass spectrometry results, the glycation modification sites of apo(a) are mainly concentrated on the kringle IV domain, whereas only a few glycation modification sites are distributed in other domains. Phenotypic experiments confirmed that glycated apo(a) and glycated apo(a)-kIV can consistently induce inflammatory factor expression and RAGE pathway activation. In a diabetic mouse model with hindlimb ischemia, intraperitoneal injection of glycated apo(a) and glycated apo(a)-kIV, respectively, resulted in a substantial inhibition of angiogenesis. Further studies have demonstrated that glycated apo(a) and glycated apo(a)-kIV promoted the expression of adhesion molecules, decreased the activities of eNOS and production of NO, and inhibited endothelial proliferation, migration, and tubular formation. Glycated apo(a) and glycated apo(a)-kIV induced endothelial dysfunction mainly through up-regulation of nuclear co-repressor NR0B1, which binds and inhibits the transcriptional activity of cardiovascular protective nuclear receptors such as LXR, NR4A1, and estrogen receptor.

### Subnormal high-density lipoprotein cholesterol level and high-density lipoprotein dysfunction

Reduced HDL-C in serum is one of the typical manifestations of diabetic dyslipidemia. The relationship between serum levels of HDL-C and coronary collateral formation remains controversial. In patients with stable CAD, one study showed that decreased HDL-C levels predicted poor coronary collateralization (38), but such results were not replicated in other studies (35, 39). These observations support a notion that HDL functionality rather than quantity alone may more reliably reflect its overall properties and has a better clinical relevance (40).

High-density lipoprotein particle is composed of an outer layer of apolipoproteins and phospholipids, surrounding a core of esterified cholesterol, and has pluripotent effects. It primarily mediates reverse cholesterol transport by carrying cholesterol from peripheral tissues to the liver for metabolism and excretion. In addition to its antioxidative, anti-inflammatory, antithrombotic and anti-apoptotic features, HDL itself has proangiogenic properties and regulates ischemia-induced angiogenesis in multiple ways (41). Cholesterol efflux capacity of HDL (HDL-CEC) is essential in maintaining cholesterol balance in endothelial cells, and it regulates angiogenesis *via* modulation of lipid rafts and VEGF receptor (VEGFR)-2 signaling (42). Large cohort studies and meta-analysis indicated that elevated HDL-CEC was associated with favorable clinical outcomes independent of circulating HDL-C levels (43). A case-control study reported a higher HDL-CEC in chronic total occlusion patients with good coronary collaterals compared to those with poor collaterals, and high HDL-CEC predicted the presence of good coronary collaterals (44). The degree of coronary collateralization from the contra-lateral vessel (usually *via* connections of the epicardial surface or intraventricular septum) was often visually estimated using the Rentrop grading system (45): 0 = no visible filling of any collateral channel; 1 = filling of side branches of the artery to be perfused by collateral vessels without visualization of epicardial segment; 2 = partially filling of the epicardial artery by collateral vessels; 3 = complete filling of the epicardial artery by collateral vessels. Patients were categorized into poor (grade 0 or 1) or good (grade 2 or 3) coronary collateralization group. This angiographic assessment of coronary collaterals is routinely applied in clinical practice. Wang et al. found that HDL-CEC correlated closely with angiographic Rentrop collateral score in non-diabetic patients, whereas HDL-CEC was impaired in patients with T2DM irrespective of collateralization status. Furthermore, this finding is supported by *in vitro* experimental results, showing that although HDL isolated from non-diabetics with poor collaterals had significantly greater potential in promoting endothelial tubular formation in Matrigel compared to HDL isolated from those with good collateralization, the proangiogenic capacity of HDL isolated from diabetic patients was markedly impaired which was not influenced by collateral conditions (46). These results imply that well-functioning HDL is biologically cardioprotective, contributing to coronary collateral formation. Nevertheless, the functional capacity of HDL is severely compromised in type 2 diabetic patients with CAD.

Glycation and oxidative modification are key underlying mechanisms that lead to HDL dysfunction and transforms the lipoprotein into a proinflammatory protein under diabetic conditions (47). Shen et al found that relative intensity of glycation of apoA-I (a predominant protein moiety in HDL) correlated positively, while HDL-associated paraoxonase (PON) 1 and PON3 activities inversely, with the severity of coronary atherosclerosis (48), and was related to decreased lecithin: cholesterol acyl transferase (LCAT) activity and plaque progression in type 2 diabetic patients undergoing percutaneous coronary intervention (49). Similarly, abundance of apo A-IV glycation was also correlated with the presence and severity of CAD in patients with T2DM. Glycosylated apo A-IV induces pro-inflammatory response *in vitro* and increases the expression of tumor necrosis factor (TNF) -α and adhesion molecules by the nuclear receptor NR4A3, thereby promoting atherosclerosis in apo E-/- mice (50). Recently, the deleterious effects of apo A-I and apo A-IV glycation on vessel growth in diabetes were assessed. In a diabetic hindlimb ischemia mouse model, blood reperfusion was determined by laser Doppler perfusion imaging after treatment with intraperitoneal injection of glycated apo A-I or glycated apo A-IV, and the gastrocnemius and soleus muscles were collected for pathological analysis and molecular biology evaluation. The results showed that both glycated apo A-I and glycated apo A-IV induced inflammatory reactions in endothelial cells and decreased new vessel growth. Further mechanistical studies revealed that glycated apo A-I skewed macrophage polarization toward M1 phenotype by activating SHP2, whereas glycated apo A-IV down-regulated cardiovascular protective nuclear receptor NR4A1 expression (51), all of which are recognized as important steps to the inhibition of angiogenesis. These findings point to a notion that HDL dysfunction and subnormal HDL-C levels act synergistically to decrease collateral formation in T2DM patients with CAD.

## Clinical relevance

**FIGURE 1:**
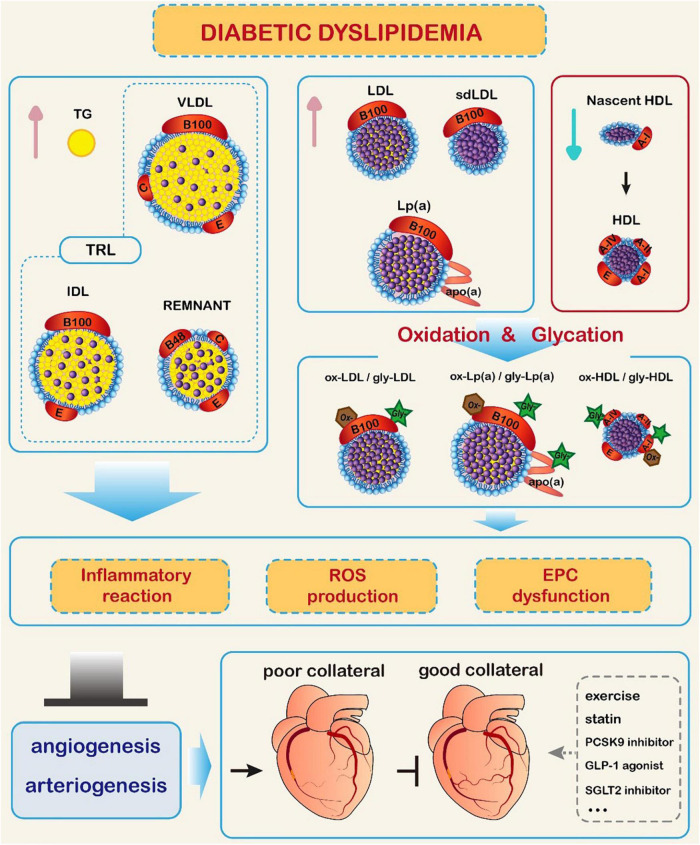
Impact of diabetic dyslipidemia on coronary collateral formation. In diabetic conditions, triglyceride (TG) and TG-rich lipoproteins (TRL) levels are increased, high-density lipoprotein cholesterol (HDL-C) level is reduced, and low-density lipoprotein cholesterol (LDL-C) level is elevated with predominance of small dense LDL-C (sdLDL-C). Meanwhile, glycation and oxidative modification of lipoprotein particles occur, promoting inflammatory reaction, production of reactive oxide species (ROS), and endothelial progenitor cell (EPC) dysfunction. These changes hamper collateral formation through inhibiting the process of angiogenesis and arteriogenesis. Exercise, lipid-lowering therapy, and antidiabetic agents may improve coronary collateral formation. 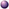
Represents cholesterol esters. GLP-1: glucagon-like peptide-1; IDL: intermediate density lipoprotein; Lp(a): lipoprotein (a); PCSK9: proprotein convertase subtilisin/kexin type 9; SGLT2: sodium-glucose cotransporter 2; VLDL: very low-density lipoprotein.

Since diabetic dyslipidemia hampers collateral vessel growth through inhibiting angiogenesis/arteriogenesis in its specific manner and coronary collateralization is of important clinical significance, the choice between the available management options for T2DM patients with CAD should account for its effect on collateral formation (Figure 1).

In terms of non-pharmacological intervention, intensive lifestyle modification (including living habitat change, exercise, and diet) exerts beneficial impact on homeostasis, lipid profiles and coronary collateral circulation (52), thus should be the main initial strategy. Cessation of cigarette smoking is proven to decrease inflammatory response, increase the number and function of EPCs, and improve VEGF activities, which are beneficial for new vessel growth (53). Regular physical exercise improves lipid profile (54), and augments myocardial oxygen demand and blood flow, acting as a driving force for arteriogenesis, which helps in coronary collateral formation in patients with stable CAD, exceeding the effect of any drug treatment (55). Similarly, optimal blood pressure control (especially diastolic blood pressure) is crucial in achieving maximal coronary collateral flow (56, 57). While dietary quality is important for overall health, the total daily caloric intake *per se* should be a key determinant of hyperlipidemia in which a hypocaloric plan is favorable for reducing overweight and improving lipid profile and insulin sensitivity (58).

Hypertriglyceridemia should be treated to eliminate residual cardiovascular risk. Fibrates, a putative agonist ligand for peroxisome proliferator activated receptor-alpha, reduce the secretion of very-low-density lipoprotein (VLDL)-triglyceride, enhance removal of LDL, and increase HDL-C levels (59). High-dose of omega-3 polyunsaturated fatty acids (PUFA), mainly eicosapentaenoic acid (EPA) and docosahexaenoic acid (DHA), is a worthwhile add-on treatment, especially in statin-treated patients with T2DM and CAD in whom triglyceride levels remain elevated (60). In addition, PUFA could attenuate inflammation, improve endothelial function, and decrease thrombus formation (61). The REDUCE-IT trial evaluated a highly purified EPA preparation (4 g/day) in patients with hypertriglyceridemia and high cardiovascular risk. The results were extraordinary, as there was a 25% relative reduction in cardiovascular events, total coronary revascularization as well as plaque burden (62). However, the STRENGTH trial failed to obtain similar favorable results by treatment with EPA/DHA preparation, and in contrast, it was associated with a slightly higher rate of atrial fibrillation (63). This discrepancy may be explained by different study design and various degrees of change in triglyceride-rich lipids as well as differential effects of EPA and DHA on membrane structure, inflammatory biomarkers, endothelial function, and tissue distribution (64). A recent study demonstrated a negative correlation between peri-coronary adipose tissue attenuation assessed by CT angiography and treatment with PUFA, suggesting a lower extent of coronary inflammation (65, 66). Adipokine C1q tumor-necrosis factor-related protein (CTRP) 1 has been shown to be involved in inflammatory reaction and disease development (67). Elevated circulating CTRP1 was associated with poor coronary collateralization in T2DM patients with stable angina pectoris. Notably, stimulation of EPCs with CTRP1 decreases both cord length and branch point number and VEGFR-2 levels (68). Whether the beneficial effect of fibrates alone or in combination with PUFA on collateral formation *via* affecting adipokines in T2DM patients with CAD merits further confirmation.

Cholesterol-lowering therapy is the mainstay in primary and secondary prevention of cardiovascular diseases (69, 70). Statins effectively decrease serum LDL-C and sdLDL-C while increasing HDL-C levels and reduce the susceptibility of apo B of LDL to undergo oxidation and glycation. They also display significant anti-inflammatory properties and improve endothelial function (so-called pleiotropic effects) (71). Robust evidence supports the fact that use of statins enhances angiogenesis as well as arteriogenesis independent of a cholesterol-lowering mechanism (72, 73). Collateral formation benefits from statin treatment in T2DM patients with CAD, due partly to reduced apoptosis and decreased release of soluble VEGFR-1 induced by proinflammatory cytokines (74, 75). Proprotein convertase subtilisin/kexin type 9 (PCSK9) inhibitors have been increasingly used in the management of dyslipidemia in individuals with T2DM (76, 77). The results of *post-hoc* subgroup analysis of randomized clinical trials indicated that alirocumab and evolocumab significantly reduced circulating LDL-C and Lp(a), and increased HDL-C, without affecting glycemic levels in patients with T2DM (78, 79). Current data concerning PCSK9 inhibitors on collateral formation are scarce. An *in vivo* study demonstrated a proangiogenic activity of evolocumab through promoting cell proliferation, migration, tubulogenesis, and VEGF secretion (80). Given their unambiguous lipid-lowering properties, such a specific role of PCSK9 inhibitors for neo-angiogenesis should be clinically attractive.

Several new hypoglycemic agents, such as glucagon-like peptide-1 (GLP-1) receptor agonists and sodium-glucose cotransporter-2 (SGLT2) inhibitors, have been shown to favorably affect lipoprotein metabolism (81, 82). Nevertheless, further studies are needed to examine if they can improve collateral formation especially for type 2 diabetic patients with CAD.

## Conclusion

In type 2 diabetic patients with CAD, the role of hypertriglyceridemia in collateral formation is not clear likely due to the concomitant changes in other lipoproteins. Elevated circulating cholesterol and Lp(a) and their glycoxidative modification hamper the process of new vessel growth. Subnormal HDL-C levels and, more importantly, deficient HDL function may act synergistically to decrease collateral formation. The choice between the available management options should account for its effect on coronary collateralization. In the future, much more research needs to be done to focus on the benefits of innovative lipid-modifying strategies, including use of PCSK9 and new triglyceride- and Lp(a)-lowering treatment, anti-diabetic agents as well as therapeutic normalization of attenuated proangiogenic and antiatherogenic HDL function, in the improvement of coronary collateral formation and clinical outcome. Novel information as such should add new knowledge on coronary pathophysiology and provide useful guidance of patient care for clinicians.

## Author contributions

YS and FD wrote the manuscript, substantially contributed to discussion of the content, and edited the manuscript. YD, XW, and YW researched data for the manuscript. RZ and LL substantially contributed to discussion of the content and reviewed the manuscript. WS reviewed the manuscript before submission. All authors read and approved the final manuscript.

## Abbreviations

AGE: advanced glycation end product
Apo: apolipoprotein
CAD: coronary artery disease
CEC: cholesterol efflux capacity
CETP: cholesterol ester transfer protein
CTRP: C1q tumor necrosis factor related protein
DPP4: dipeptidyl peptidase-4
eNOS: endothelial nitric oxide synthase
FGF: fibroblast growth factor
GLP-1: glucagon-like peptide-1
HDL: high-density lipoprotein
HDL-C: HDL cholesterol
HGF: hepatocyte growth factor
HIF: hypoxia-inducible factor
LCAT: lecithin cholesterol acyl transferase
LDL: low-density lipoprotein
LDL-C: low-density lipoprotein cholesterol
Lp(a): lipoprotein(a)
MCP: monocyte chemotactic protein
NO: nitric oxide
oxLDL-C: oxidized low-density lipoprotein cholesterol
PCSK9: proprotein convertase subtilisin/kexin type 9
PON: paraoxonase
PUFA: polyunsaturated fatty acid
RAGE: receptor for advanced glycation end product
ROS: reactive oxide spices
sdLDL-C: small dense low-density lipoprotein cholesterol
SGLT2: sodium-glucose co-transporter-2
T2DM: type 2 diabetes mellitus
TG/HDL-C: triglyceride/high-density lipoprotein cholesterol
TRL: triglyceride-rich lipoprotein
TyG index: triglyceride-glucose index
VEGF: vascular endothelial growth factor
VEGFR: VEGF receptor
VLDL: very low density lipoprotein.
